# Secretome of adipose-derived mesenchymal stem cells promotes skeletal muscle regeneration through synergistic action of extracellular vesicle cargo and soluble proteins

**DOI:** 10.1186/s13287-019-1213-1

**Published:** 2019-04-05

**Authors:** Robert Mitchell, Ben Mellows, Jonathan Sheard, Manuela Antonioli, Oliver Kretz, David Chambers, Marie-Theres Zeuner, James E. Tomkins, Bernd Denecke, Luca Musante, Barbara Joch, Florence Debacq-Chainiaux, Harry Holthofer, Steve Ray, Tobias B. Huber, Joern Dengjel, Paolo De Coppi, Darius Widera, Ketan Patel

**Affiliations:** 1 School of Biological Sciences, University of Reading, Reading, UK; 2 Stem Cell Biology and Regenerative Biology Group, School of Pharmacy, University of Reading, Reading, UK; 3 Sheard BioTech Ltd, 20-22 Wenlock Road, London, N1 7GU UK; 4 I.N.M.I. L Spallanzani IRCCS, Rome, Italy; 5 Department of Medicine III, Faculty of Medicine University Medical Center Hamburg-Eppendorf, Hamburg, Germany; 6 Renal Division, Medical Centre, Faculty of Medicine, University of Freiburg, Freiburg, Germany; 7 Department of Neuroanatomy, Institute for Anatomy and Cell Biology, Faculty of Medicine, University of Freiburg, Freiburg, Germany; 8 Wolfson Centre for Age-Related Diseases, King’s College, London, UK; 9 Interdisciplinary Centre for Clinical Research Aachen, RWTH Aachen University, Aachen, Germany; 10 Centre for Bioanalytical Sciences (CBAS), Dublin City University, Dublin, Ireland; 11 URBC, Namur Research Institute for Life Science (NARILIS), University of Namur, Namur, Belgium; 12 FRIAS Freiburg Institute for Advanced Studies, University of Freiburg, Freiburg, Germany; 13 Micregen, Alderley Edge, Manchester, UK; 14 BIOSS Centre for Biological Signalling Studies and Centre for Systems Biology (ZBSA), Albert-Ludwigs-University, Freiburg, Germany; 15 Department of Biology, University of Fribourg, Fribourg, Switzerland; 16 Stem Cells & Regenerative Medicine Section, UCL Great Ormond Street Institute of Child Health, London, UK

**Keywords:** Adipose-derived mesenchymal stem cell, Secretome, Extracellular vesicles, Muscle, Regeneration, Inflammation, microRNA, Proteomic

## Abstract

**Background:**

The mechanisms underpinning the regenerative capabilities of mesenchymal stem cells (MSC) were originally thought to reside in their ability to recognise damaged tissue and to differentiate into specific cell types that would replace defective cells. However, recent work has shown that molecules produced by MSCs (secretome), particularly those packaged in extracellular vesicles (EVs), rather than the cells themselves are responsible for tissue repair.

**Methods:**

Here we have produced a secretome from adipose-derived mesenchymal stem cells (ADSC) that is free of exogenous molecules by incubation within a saline solution. Various in vitro models were used to evaluate the effects of the secretome on cellular processes that promote tissue regeneration. A cardiotoxin-induced skeletal muscle injury model was used to test the regenerative effects of the whole secretome or isolated extracellular vesicle fraction in vivo. This was followed by bioinformatic analysis of the components of the protein and miRNA content of the secretome and finally compared to a secretome generated from a secondary stem cell source.

**Results:**

Here we have demonstrated that the secretome from adipose-derived mesenchymal stem cells shows robust effects on cellular processes that promote tissue regeneration. Furthermore, we show that the whole ADSC secretome is capable of enhancing the rate of skeletal muscle regeneration following acute damage.

We assessed the efficacy of the total secretome compared with the extracellular vesicle fraction on a number of assays that inform on tissue regeneration and demonstrate that both fractions affect different aspects of the process in vitro and in vivo.

Our in vitro, in vivo*,* and bioinformatic results show that factors that promote regeneration are distributed both within extracellular vesicles and the soluble fraction of the secretome.

**Conclusions:**

Taken together, our study implies that extracellular vesicles and soluble molecules within ADSC secretome act in a synergistic manner to promote muscle generation.

**Electronic supplementary material:**

The online version of this article (10.1186/s13287-019-1213-1) contains supplementary material, which is available to authorized users.

## Background

Mesenchymal stem cells (MSCs) are an attractive therapeutic tool for regenerative medicine due to their capacity for self-renewal and the ability to differentiate into a variety of mesodermal lineages [1, 2]. It has been postulated that due to their inherent multipotency, MSCs are able to directly integrate into diseased organs and tissues [3]. In this context, transplantation of MSCs has been reported to be beneficial in cartilage regeneration [4], regeneration of bone tissue [5] and in acute and chronic models of muscle degeneration [6, 7]. However, although these and other studies reported an abrogation of pathology, the level of MSC engraftment into the diseased organs is often negligible (less than 1%) [8–10]. This led to an alternative hypothesis suggesting that factors produced by MSCs acting in a paracrine manner rather than the cells themselves promote tissue regeneration [11–15]. To date, the regenerative effect of factors secreted by MSCs have been investigated in many different conditions including regeneration of the heart [16], central nervous system (CNS) [17], kidney [18], muscle tissue [19] and wounds [20] suggesting that the MSC secretome may be as efficacious as the cells themselves. Many studies have also described MSCs as having immune-privilege potential, with several MSC products being approved for clinical application [21, 22].

Stem cells are known to interact and actively communicate with their surrounding microenvironment [23] via the secretion of cytokines and growth factors including but not limited to insulin-like growth factor-1 (IGF-1) [24], hepatocyte growth factor (HGF) [3], interleukins [25] and vascular endothelial growth factor (VEGF) [26]. These molecules regulate a variety of different cell activities essential in tissue regeneration, such as proliferation, angiogenesis and the modulation of inflammation [21, 27, 28]. Proteins secreted by stem cells are found either as free entities or within membrane particles such as exosomes and microvesicles (MV), collectively known as extracellular vesicles (EVs). Originally considered to be cellular debris, EVs are now understood to play a vital role in cell-to-cell communication [29, 30] and may mediate the immunomodulatory effects of MSCs [31]. In addition to proteins, mRNA, miRNA and organelles are packaged within EVs [32–34] which collectively constitute the stem cell secretome.

Human adipose tissue is easily accessible and a source of multipotent MSCs that can be applied for autologous transplantation [35]. In addition to mediating bone and cartilage regeneration, ADSCs have been reported to positively modulate endogenous regeneration of muscle [36, 37].

Recently, we reported that the secretome from human amniotic fluid stem cells (AFSCs) has anti-inflammatory properties, promotes stem cell proliferation, protects against cellular senescence and facilitates tissue regeneration in a cardiotoxin (CTX)-induced model of muscle degeneration [38].

In the present study, we wanted to investigate if the application of the same isolation method would produce an ADSC secretome that has similar regenerative potential and if the EV fraction was solely responsible for these effects. Furthermore, we have taken a bioinformatic approach to compare the protein and molecular components of the ADSC secretome with the secretome of AFSCs that also promotes muscle regeneration.

We show that the secretome produced using our protocol contained mostly EVs, rich in miRNA and not mRNA. Assessing the biological activity of the whole ADSC secretome, we demonstrate that similar to AFSC secretome, it is capable of promoting cell proliferation and migration.

Importantly, we show that the whole secretome and the EV fraction have a differing impact on the ability to protect against senescence and in a molecular model for inflammation based on nuclear translocation of NF-κB.

In order to comparatively assess the biological activity of both fractions in vivo, we used the CTX model of acute muscle injury. Both fractions increased the cross-sectional area of newly regenerated muscle fibres and reduced the numbers of infiltrating macrophages with the EV fraction resulting in significantly stronger effects. However, we detected significant differences between the impact of the whole secretome and the EV fraction on the muscle stem cells during regeneration.

Mass spectrometry was performed to determine the ADSC-EV protein cargo and proteins present in the soluble fraction alone. Bioinformatic analysis revealed 96 proteins exclusively present in the soluble and 301 in the EV fraction of the secretome.

Analysis of the miRNA cargo of the EVs revealed a broad range of miRNAs present in samples from three individual donors targeting pathways regulating the stress response, differentiation, proliferation and immune modulation.

Our comparison of ADSC and AFSC secretomes revealed 108 mutually exclusive protein hits in the EV fractions of both secretomes and 50 proteins present in both soluble fractions. A comparison of the miRNA cargo of EVs from ADSCs and AFSCs revealed 519 mutually exclusive miRNAs and only 47 miRNA exclusively present in ADSC EVs.

In conclusion, our results indicate that the EV-cargo is mainly but not exclusively responsible for the regenerative action of the ADSC secretome in CTX-induced muscle injury model. Moreover, our data implicates that the beneficial effects of the paracrine factors are a result of a synergistic action of the miRNA and protein cargo of the EV fraction and the soluble proteins within the total secretome.

## Materials and methods

### Cell culture

Human ADSCs (Life Technologies, Cat # 510070) were cultured in MesenPro RS™ (Life Technologies, 12746-012) supplemented with 1% l-glutamine (Life Technologies, 25030-081) and 1% penicillin streptomycin (Life Technologies, 15070-063). All experiments were carried out with cells at passage 6 or below.

Human lung fibroblasts (IMR-90) were used at 50% Hayflick limit (27–32 population doublings) and cultured in αMEM supplemented with 10% FBS (Life Technologies 10500-064), 1% l-glutamine and 1% penicillin streptomycin.

Human U251-MG Glioblastoma cells (U251) (Cell Line Service) were cultured in high glucose DMEM supplemented with 10% FBS, 1% l-glutamine and 1% penicillin streptomycin.

A10 rat smooth muscle cells were cultured in high glucose DMEM supplemented with 10% FBS and 1% l-glutamine.

C2C12 cells were cultured in DMEM supplemented with 10% FBS, 1% l-glutamine and 1% penicillin streptomycin and differentiated in DMEM supplemented with 2% horse serum, 1% l-glutamine and 1% penicillin streptomycin.

NHDF Fibroblast cells (Lonza, Cat # CC-2511) were cultured in DMEM supplemented with 10% FBS, 1% l-glutamine and 1% penicillin streptomycin.

### ADSC secretome generation and extracellular vesicle (EV) isolation

ADSCs cultivated as adherent monolayer were harvested via enzymatic dissociation (TrypLE™ Select, Life Technologies, 12563-011) and washed 3× in sterile 1× PBS (Life Technologies, 10010-056) with centrifugation at 300*g* for 5 min at room temperature (RT) between washes, before aliquoting 1 × 10^6^ cells per tube. Each aliquot was pelleted and covered with 400 μL fresh sterile PBS and maintained at room temperature for 24 h. Thereafter, the supernatant was aspirated, pooled, sterile filtered through a 0.2-μm syringe filter (Pall Life Sciences, 4652) and centrifuged at 2000*g* for 20 min at RT (and hereafter referred to as ‘total ADSC secretome’). The whole secretome was ultracentrifuged at 200,000*g* for 18 h at 4 °C. The supernatant was aspirated (soluble fraction) and pellets re-suspended in PBS (40 μL/1 × 10^6^ cells) to produce the EV fraction.

### TEM and EV size analysis

A single drop of re-suspended EV pellet was placed onto parafilm and adsorbed onto carbon-coated copper-meshed grids by placing the latter onto the drops for 5 min. The samples were fixed with 1% glutaraldehyde, washed four times for 30 s and negatively contrasted using 1% uranyl acetate. Grids were air dried and analysed using a Zeiss 906 transmission microscope. EV size was quantified by manually measuring the diameter of EV populations from three separate batches of complete secretome on Axiovision image analysis software (version 4.7). Protein content of the whole secretome and EV fraction was analysed by SDS PAGE followed by silver staining. Briefly, 6 μg of denatured protein was resolved on a 4–12% SDS PAGE gel prior to processing with the SilverXpress® silver stain kit (Life Technologies LC6100) and imaged using Syngene G:BOX using GeneSys software.

### EV concentration and size analysis using nanoparticle tracking analysis

The concentration and the size of EVs within the whole secretome was assessed using nanoparticle tracking analysis (NTA) as described in [39] using an NS500 instrument (Nanosight Ltd., Amesbury, UK).

### Assessment of EV uptake by IMR-90 cells

ADSC EV were labelled with fluorescent dye PKH67 (Sigma Aldrich MIDI67) by adding 40 μL of EV fraction (EV from 1 × 10^6^ cells) to PKH67 dye solution followed by incubation for 5 min at room temperature before being ultracentrifuged at 200,000*g* for 18 h at 4 °C. Following centrifugation, the supernatant was aspirated and EV pellet resuspended in 100 μL PBS. For the cellular uptake assays, IMR90 cells at 40% confluency were washed 3× with DMEM and incubated with 5 μM CellTracker™ Red (Invitrogen CMTPX) for 30 min at 37 °C, 5% CO_2_. PKH67-stained EVs were added to CellTracker™ Red-stained IMR90 cells and incubated for 3 h at 37 °C, 5% CO_2_. The cells were fixed in 4% paraformaldehyde for 15 min at room temperature, washed 3× in PBS and sections mounted using mounting medium containing 2.5 μg/mL 4’,6-diamidino-2-phenylindole (DAPI) for nuclear visualisation. Confocal images were captured using the Nikon A1-R inverted confocal microscope with the Nikon Plan Apo VC 100x DIC N2 optic lens, running NIS Elements AR.

### Flow cytometry

ADSCs were fixed in 4% paraformaldehyde at RT for 20 min and non-specific binding blocked with 5% FBS. Antibodies (multipotency markers: CD44 (Millipore, CS208200 1:20), CD73 (BD Biosciences, 551123 1:20), CD90 (BD Biosciences, 554895 1:10) and non-MSC markers: CD34 (Millipore CBL555F 1:20) and CD45 (BD Biosciences, 554875 1:10)) were incubated for 1 h at 4 °C. Ten thousand events were profiled by flow cytometry (BD Accuri C6 Flow Cytometer, C-sampler) followed by data analysis in FlowJo, LLC v10.

### Multipotency assessment

For assessment of adipogenic and osteogenic potential after secretome collection, 4000 cells/cm^2^ were plated and cultured to 95% confluency before growth media were replaced with either adipogenic (R&D Systems CCM007 and CCM011) or osteogenic (Life Technologies A10069-01 and A10066-01) differentiation media for 21 days. Adipogenesis or osteogenesis was determined by Oil Red O (Sigma Aldrich O0625) or Alizarin Red S (Sigma Aldrich A5533) staining respectively. Bright field images were captured immediately after staining. Chondrogenic potential was assessed by resuspending 1 × 10^6^ ADSC cells in a 1.5-mL tube and allowing the formation of a pellet by gravity. The pelleted cells were incubated in differentiation media (Life Technologies A10069-01 and A10064-01) for 21 days with a media change every 3 days. Thereafter, the ADSC pellet was embedded in tissue freezing medium and cryosectioned at 13 μm. Chondrogenic cells were identified with 0.1 mg/mL Alcian Blue staining solution (Sigma Aldrich A5268) [40].

### Proliferation assay

C2C12 cells were seeded into 6-well cell culture plates (Sarstedt) at 3000 cells/cm^2^ and allowed to adhere for 24 h. Two percent whole secretome (*v*/*v*) in growth media was added to the cells followed by incubation for 48 h and manual cell counting using a haemocytometer.

### Differentiation assay

C2C12 cells were seeded into 6-well plates (Sarstedt) at 3000 cells/cm^2^ and cultured in growth media until they reached 95% confluency, where the growth media was replaced with differentiation media supplemented with 2% whole secretome (*v*/*v*). The cells were further cultured for 72 h, fixed using 2% PFA and processed for immunocytochemical staining using an antibody against Myosin Heavy Chain (Millipore, 05-716, clone A4.1025 1:1).

### Wound closure (migration) assay

A10 rat smooth muscle cells were seeded into a 24-well plate (Sarstedt) at 36,000 cells/cm^2^ and incubated overnight in standard growth media. Following incubation, scratches were made with a p20 pipette tip vertically through the well. Media within each well was removed, and the cells were washed with 1 mL pre-warmed PBS. The PBS was replaced with complete media supplemented with 2% (*v*/*v*) vehicle control (PBS) or 2% (*v*/*v*) whole secretome. Migration was assessed using the Nikon TiE imaging system where images were gathered every 30 min over a 16-h period. Analysis was performed on images taken at 0 h compared to those gathered at 6 h (where a ~ 50% gap closure was observed). Gap area at 6 h was quantified and presented as a percentage closure relative to that at 0 h.

### Senescence assay

Stress-induced senescence was induced in IMR-90 cells using H_2_O_2_ as previously described [41]. Briefly, 50,000 IMR-90 cells were cultured in a 12-well plate until 70% confluent, at which point 10% secretome or 2% EV (from 1 × 10^6^ cells) (*v*/*v*) was introduced in growth medium. Cells were incubated for 24 h before being exposed to 100 μM H_2_O_2_ in growth medium for 2 h. Thereafter, cells were washed twice in PBS, maintained in growth media for 48 h, passaged and plated in a 24-well plate at 800 cells/well before staining for β-galactosidase activity using X-Gal. Analysis of senescent cells was carried out by manually counting the proportion of cells stained blue on an inverted epifluorescence microscope (Zeiss A1).

### Ethical approval

The experiments were performed under a project licence from the United Kingdom Home Office in agreement with the Animals (Scientific Procedures) Act 1986. The University of Reading Animal Care and Ethical Review Committee approved all procedures.

### Animal maintenance

Healthy male wild-type C57BL/6 mice (2–3 month) were maintained in accordance with the Animals (Scientific Procedures) Act 1986 (UK) under a project licence from the United Kingdom (UK) Home Office. Mice were housed under standard environmental conditions (20–22 °C, 12–12-h light–dark cycle) and were provided with food (standard pelleted diet) and water ad libitum.

### Acute skeletal muscle degeneration

Non-immunosuppressed mice were warmed for 10 min in a hot box at 40 °C prior to the intra-venous (IV) injection of 100 μL sterile 0.1 M PBS, 100 μL whole secretome or 100 μL EV (from 1 × 10^6^ cells) through the lateral tail vein (5 mice per condition). Thirty minutes later, mice were anaesthetised with 5% isofluorane and intra-muscular (IM) injected with 30 μL of 50 μM cardiotoxin I (CTX) from *Naja pallida* (Latoxan, Valence France) into the right tibialis anterior (TA) muscle. Thirty microlitre sterile PBS was injected into the left TA which served as the undamaged control muscle. Mice were then maintained for 5 days before being humanely sacrificed via schedule 1 killing. Following culling, the TA muscle was snap frozen on frozen iso-pentane before storage at − 80 °C. Thirteen-micrometre cryosections were made using a Bright OTG cryostat (OTF/AS-001/HS) for immunohistochemistry or histological examination.

### Histology, immunohistochemistry and cellular EV uptake

Acid phosphatase staining was analysed for compromised muscle fibre integrity by incubating TA cryosections in acid phosphatase buffer (HPR reagent, 0.1 M acetate buffer pH 5.0, 50 mg/mL naphthol AS-BI phosphate) for 90 min at 37 °C before being washed and counterstained with a 1:30 dilution of Harris haematoxylin for 1 min. The stained slides were mounted using hydromount.

Immunocytochemistry was performed as previously described [42] with the following antibodies: monoclonal anti-mouse Pax7 (DSHB-Pax7) (1:1), monoclonal anti-mouse Myosin heavy chain 3 (DSHB-F1.652) (1:1), Rat anti-mouse CD31 (BioRad MCA2388) (1:150), Rat anti-mouse F4-80 (BioRad MCA497R) (1:200), polyclonal rabbit anti-MyoD (Santa Cruz sc-760) (1:200), monoclonal mouse anti-CD68 (Abcam ab955) (1:200), and monoclonal mouse anti-NF-κB-P65 (F-6) (Santa Cruz sc8008) (1:200). Secondary antibodies used were Alexa Fluor 488 Goat anti-mouse IgG (Invitrogen A11029) (1:200), Alexa Fluor 594 Goat anti-Rabbit IgG (Invitrogen A11012) (1:200), and Rabbit anti-Rat HRP (DAKO P0450) (1:200). Sections were mounted using mounting medium, containing 2.5 μg/mL 4’,6-diamidino-2-phenylindole (DAPI) (Molecular Probes D1306) for nuclear visualisation.

### Sample preparation for mass spectrometry (MS) and analysis

ADSC-EV (EV) and non-EV (soluble) fractions isolated from ADSC secretome were obtained as described above. Proteins were suspended in 1× SDS-PAGE loading buffer, denaturated and reduced with 1 mM DTT for 10 min at 95 °C, then alkylated using 5.5 mM iodacetamide for 30 min at 25 °C in the dark. Proteins were resolved by SDS-PAGE using 4–12% Bis-Tris mini gradient gels. Each lane was cut into 10 equal slices and proteins therein were in-gel digested with trypsin. Peptides were dried to less than 5 μl and resuspended in 15 μl of 0.5% acetic acid for the MS analysis.

LTQ Orbitrap XL mass spectrometer coupled to an Agilent 1200 nanoflow-HPLC was used to measure peptides derived from trypsin digestion. Samples, applied directly onto self-packed HPLC-column tips of around 20 cm, were subjected to a gradient formed by solution A (0.5% acetic acid LC–MS grade in water) and by increasing organic proportion of solution B (0.5% acetic acid in 80% ACN LC–MS grade in water) within 120 min [43]. MaxQuant software [44] version 1.4.1.2 was used to identify proteins based on peptides profiles and to perform label-free quantification. Both Orbitrap and MaxQuant parameters were set as previously described [45]. iBAQ quantification values were log2-transformed, and subsequently, missing values were imputed by normal distribution. GO terms’ analyses for the entire dataset and for 269 proteins identified in all samples were performed by principal component analysis considering the enrichment of 4 categories and using a cut-off of Benjamini-Hochberg FDR lower than 0.05.

### Analysis of the miRNAs

GeneChip® miRNA 4.0 Arrays were used to analyse the miRNA content of ADSC secretome (FlashTag™ Biotin HSR RNA Labeling Kit according to the manufacturer’s instructions was used). Probe cell intensity data (CEL) from Affymetrix GeneChip® miRNA 4.0 Arrays were analysed in the Affymetrix Expression Console™ software. Normalisation was performed using the Robust Multichip Analysis (RMA) + DABG algorithm [46]. Only miRNAs calculated as present in all 3 samples were declared as generally expressed after 24 h. Focus was given to the miRNAs present with the highest signal intensities (top 50). For these top 50 miRNAs, validated target mRNAs were amalgamated using miRWalk2.0 software [47]. In the next step, GO Slim classification for biological process was performed using WebGestalt software to provide a high-level functional classification for validated target mRNAs [48]. AltAnalyze software (Version 2.1.0) was used to design the heat map showing intensity variability between biological replica (*n* = 3) for the top 50 (hierarchical clustering heat map using cosine column and row clustering, rows were normalised relative to median).

### Protein functional annotation

Proteins identified in specific fractions of the ADSC secretome were subjected to functional enrichment analysis to provide insight into the functional associations of these protein subsets. This analysis was performed independently using g:Profiler g:GOSt [49] for enrichment of biological process (BP) Gene Ontology (GO) terms. Statistical significance was calculated using the Fisher one-tailed test in combination with the default g:SCS algorithm to correct for multiple testing. Inferred electronic GO annotations were not included, and data was not subjected to hierarchical filtering. Significantly enriched GO terms were grouped using an in-house semantic ontology to aid interpretation [50].

### Statistical analysis

Statistical analysis was performed with GraphPad Prism software using unpaired Student’s *t* test or a one-way analysis of variance followed by post hoc Tukey’s test unless otherwise stated. A minimum of 95% confidence interval was used for significance; *p* values indicated on figures were *p* < 0.05 (*), *p* < 0.01 (**) or *p* < 0.001 (***). Data was presented as mean ± SEM.

## Results

### ADSC secretome contains soluble proteins and EVs with miRNA and proteins as a cargo

**Fig. 1:**
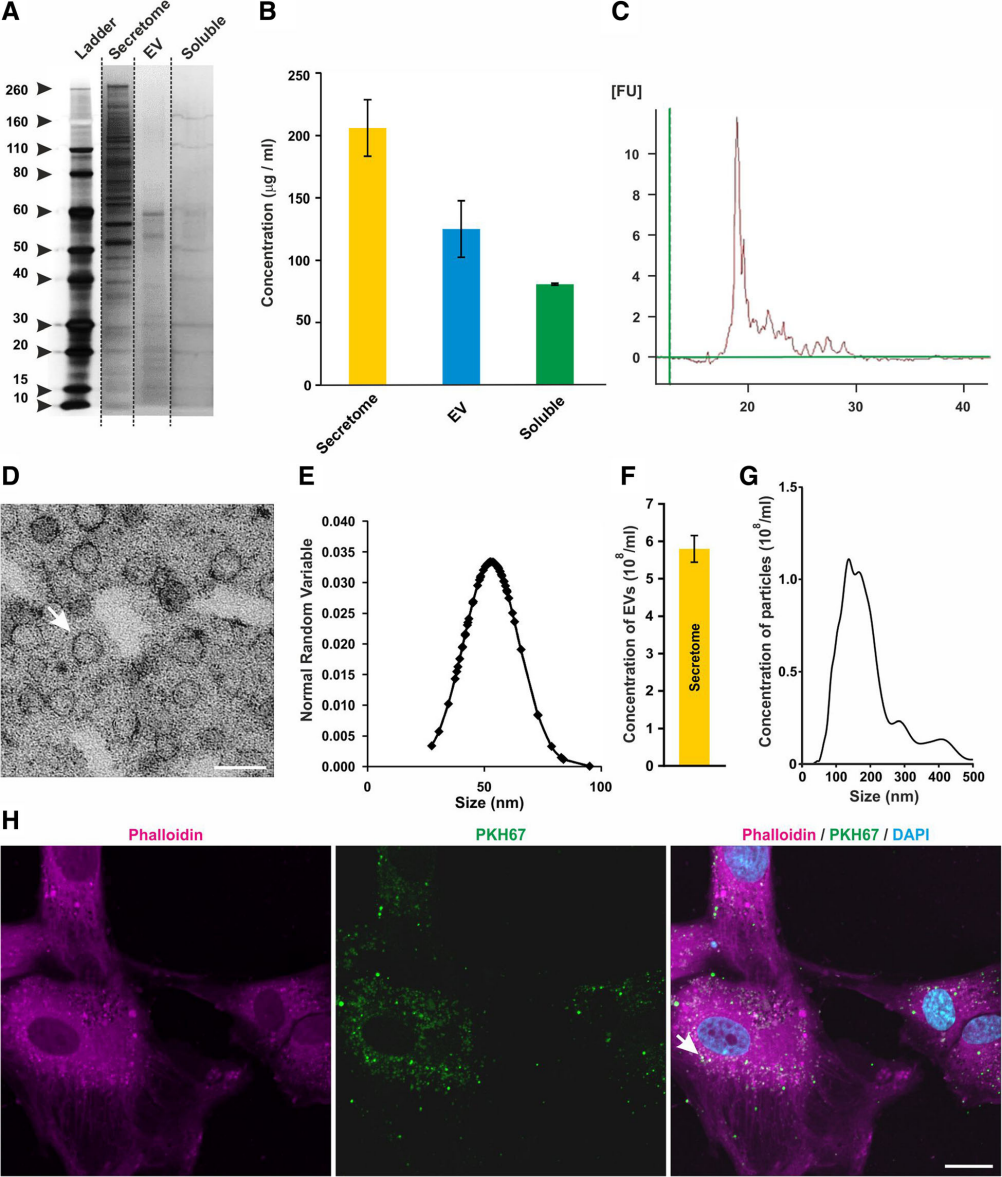
ADSC secretome contains soluble proteins and EVs with miRNA and proteins as a cargo. **a**, **b** SDS-PAGE with subsequent silver staining of the total ADSC secretome, EV fraction, and soluble fraction revealed a wide spectrum of proteins, with each fraction showing a distinctive size distribution profile. **c** Profiling the nucleic acids content using bioanalyzer PicoRNA chips showed no molecules larger than ~ 31 nucleotides. **d**–**g** The EVs were analysed using TEM and NTA revealing a size of ~ 50 nm. Scale bar: 100 nm. **h** EVs were labelled with PKH67 and incubated with IMR-90 cells. Overlap between the PKH67 signal and phalloidin-stained cytoskeleton demonstrates that ADSC-derived EVs are taken up by IMR-90 cells (white arrow). Scale bar: 20 μm

In order to visualise and quantify the protein components within the total ADSC secretome, isolated EVs (EV fraction) and EV-depleted secretome (soluble fraction), SDS-PAGE with subsequent silver staining was performed (Fig. 1a). Within the total secretome, we were able to detect a wide spectrum of proteins (10–260 kDa) with a concentration of 206.0 μg/mL corresponding to 82.4 μg/million cells (Fig. 1a, b). Analysis of the EV fraction revealed a concentration of 125.1 μg/ mL (50.0 μg/million cells) whereas 803.9 μg/ mL (32.4 μg/million cells) of total protein was detected in the soluble fraction (Fig. 1b). Notably, each of the analysed fractions showed a distinctive size distribution profile (Fig. 1a, b). Profiling the nucleic acids content in the total ADSC secretome using bioanalyzer PicoRNA chips identified the presence of small RNAs with no molecules larger than 30 nucleotides, suggesting that the RNA components are exclusively miRNAs (Fig. 1c).

### Characterisation of ADSC secretome EV

For visualisation, EVs were isolated from total ADSC secretome using ultracentrifugation and processed for TEM. Size distribution analysis revealed an average EV diameter of 52.2 nm without the presence of larger particles (Fig. 1d, e). In order to verify the size distribution and to determine the number of particles, nanoparticle tracking analysis (NTA) was applied revealing 17.4 × 10^8^ EVs/mL in a secretome derived from 2.5 × 10^6^ cells (Fig. 1f). In accordance with the TEM analysis, NTA results indicated an average EV size of 57 nm (Fig. 1g).

### ADSC-derived EV are taken up by IMR-90 cells

To monitor their uptake, ADSC-derived EVs were labelled with PKH67 and incubated with IMR-90 cells. Subsequent laser scanning microscopy and image analysis revealed that ADSC-derived EVs were able to interact and to be taken up by host cells as indicated by intracellular PKH67 signal in host cells (Fig. 1h, white arrow).

### ADSCs do not change their characteristics following secretome generation

In order to assess potentially detrimental effects that the generation of the whole secretome may have upon the ADSCs, we tested the viability, multipotency marker expression and differentiation capability of ADSCs 24 h after the secretome generation. We found a viability of 71.2% (data not shown) and detected no significant changes in the expression pattern of positive and negative MSC markers. Ninety-five percent of the surviving ADSCs after the PBS incubation period remained positive for the mesenchymal stem cell (MSC) markers CD44, CD73 and CD90 and displayed less than 1% expression for the negative MSC markers CD34 and CD45 (Additional file 1: Figure S1A-B). Furthermore, the surviving cells readily differentiated into osteogenic (Additional file 1: Figure S1C), adipogenic (Additional file 1: Figure S1D) and chondrogenic cells (Additional file 1: Figure S1E).

### ADSC secretome promotes cell proliferation, skeletal muscle differentiation and migration

**Fig. 2:**
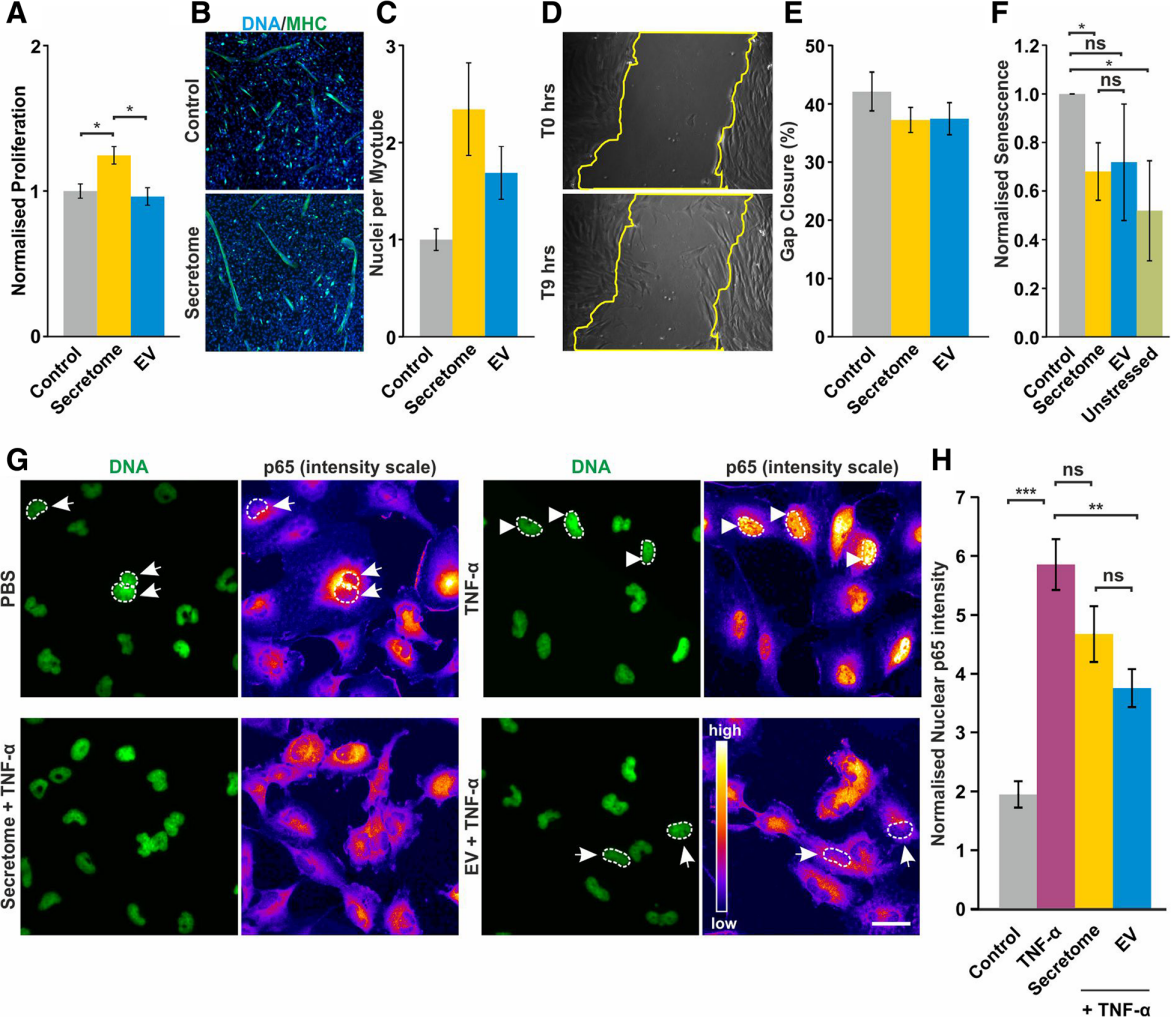
Total ADSC secretome and the EV fraction affect three hallmarks of regeneration in vitro. **a** The impact of ADSC secretome on cell proliferation was assessed using total cell number determination revealing a significant increase in proliferation after exposure of C2C12 cells to the whole secretome. **b**, **c** Immunocytochemical analysis demonstrated an increase of C2C12 cell differentiation towards myotubes after whole secretome treatment. **d**, **e** Wound healing assay was performed to assess the impact of the secretome on migration of A10 muscle cells. No significant changes in migration of A10 cells exposed to the whole secretome were observed. **f** In order to study the effects of the total secretome and ADSC-derived EVs on cellular senescence, IMR-90 cells were co-exposed to H_2_O_2_ and vehicle (PBS), whole secretome, and the EV-fraction followed by staining for β-Galactosidase activity. Analysis of the number of senescent cells revealed that both total ADSC secretome and the EV fraction protect against cellular senescence. **g**, **h** Only the EV fraction (arrows) significantly reduces levels of inflammation in the U251 cell model compared to the TNF-α exposed control cells showing high level of nuclear p65 (arrowheads). Scale bar: 20 μm. *p* < 0.05 (*), *p* < 0.01 (**) or *p* < 0.001 (***). Three separate batches of ADSC secretome and isolated EV fraction were tested

Tissue regeneration and homeostasis rely on cell proliferation, stem cell differentiation and cellular migration. Thus, we investigated the impact of the total ADSC secretome on these three hallmarks of regeneration. Total number determination of the mouse myoblast cell line C2C12 exposed for 48 h to the total secretome revealed a significant increase of cell proliferation compared to control cells (Fig. 2a). Moreover, the total secretome was able to stimulate the differentiation of C2C12 cells towards myotubes (Fig. 2b, c). To assess the effect of the total secretome on cell migration, a wound closure assay was performed, revealing no effect on migration in the presence of the secretome compared to the control (Fig. 2d, e).

### Total ADSC secretome and the EV fraction both protect against cellular senescence, but only the EV fraction reduces the levels of inflammation in U251 cells

Protection against cellular senescence was assayed by exposure of IMR-90 cells to 100 μM H_2_O_2_. Forty-eight percent of stressed cells treated with PBS showed ß-Gal activity. In contrast, exposure of IMR-90 cells to the total ADSC secretome prior to induction of stress stimulus reduced the ß-Gal activity to the level of unstressed control cells (Fig. 2f). Notably, co-treatment with 100 μM H_2_O_2_ and the EV fraction resulted in a similar, yet non-significant reduction of the proportion of ß-Gal-positive cells (Fig. 2f).

In order to comparatively assess the anti-inflammatory potential of ADSC secretome and enriched ADSCs EV, we investigated the effect of both fractions on the TNF-α-induced nuclear translocation of the NF-κB subunit p65 in U251 cells (Fig. 2g, h). Notably, the level of nuclear NF-κB p65 was significantly increased by TNF-α treatment compared to control cells (Fig. 2g, h). Exposure of TNF-α stimulated cells to the total secretome fraction led to a slight and non-significant reduction of nuclear p65 whereas the EV fraction resulted in a significantly reduced amount of p65 in the nuclei (Fig. 2g, h).

### Regenerative effect of ADSC secretome is not replicated in control fibroblast secretome

To investigate if the regenerative effects observed are indicative to ADSC or can be observed via treatment with any whole secretome, we generated a whole secretome from a fibroblast cell source under identical conditions to that of the ADSC secretome and tested it under many of the same in vitro assays as the ADSC secretome. The fibroblast secretome contained a much lower total protein concentration than the ADSC whole secretome (46 μg/mL compared to 206 μg/mL) (Additional file 2: Figure S2A). NTA of the fibroblast vesicle content identified a similarly lower vesicle concentration (2.39 × 10^8^ vesicles/mL), whilst having a similar size distribution (137 nm mean size) to the ADSC EV (Additional file 2: Figure S2B-C).

Treating the C2C12 cells with the fibroblast secretome for 48 h saw no significant increase in cell proliferation (Additional file 2: Figure S2D), and there was also no effect on the rate of cell fusion, a measure of myogenic differentiation (Additional file 2: Figure S2E). Finally, carrying out a migration assay saw a slight, but non-significant increase in gap closure (Additional file 2: Figure S2F).

### Total ADSC secretome and the isolated EV fraction modulate different aspects of in vivo regeneration in the CTX-induced acute muscle injury model

**Fig. 3:**
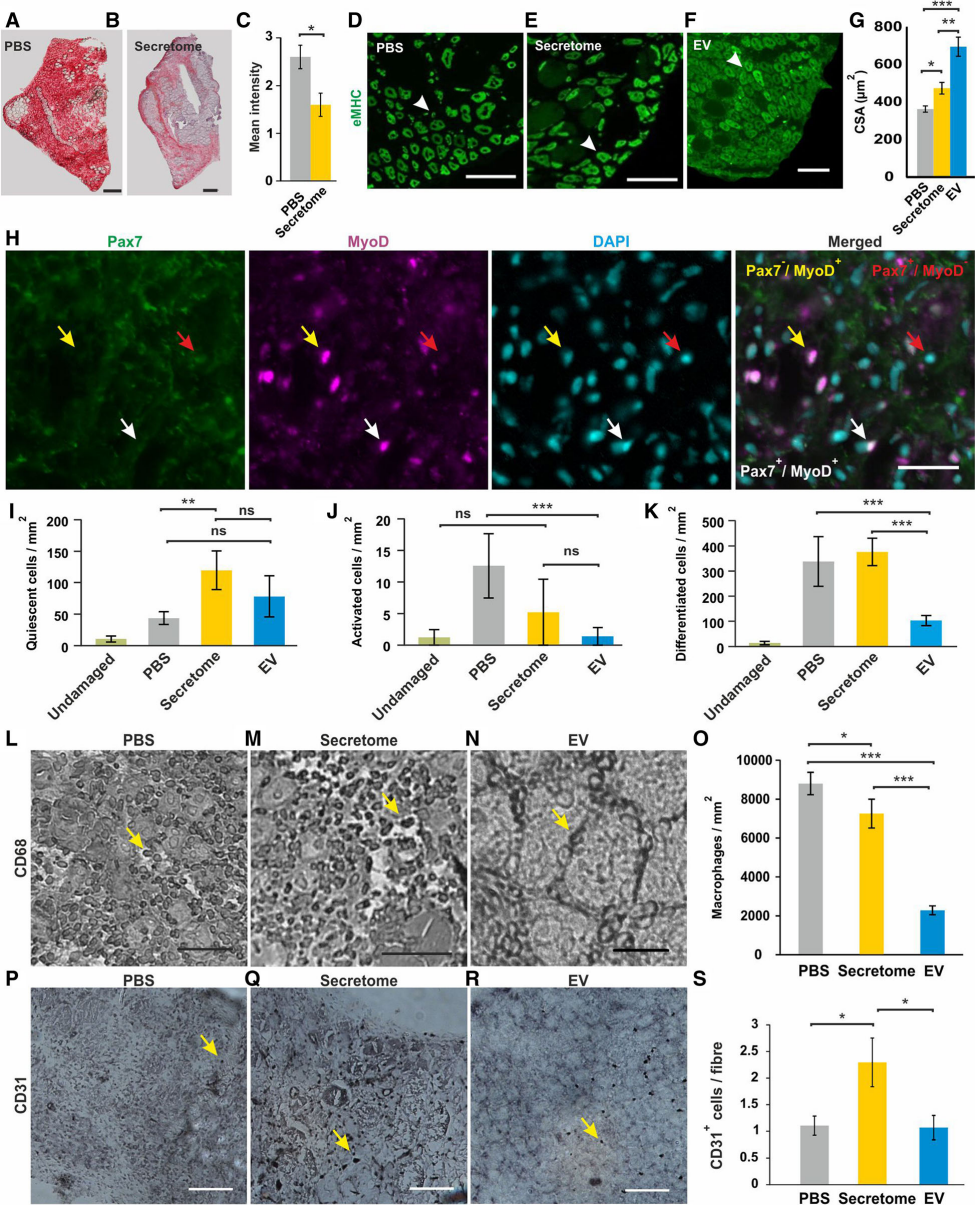
Total ADSC secretome and its EV fraction modulate different aspects of in vivo skeletal muscle regeneration. The regenerative effect of an intravenous injection of either total ADSC secretome or its isolated EV fraction was assessed in the acute skeletal muscle degeneration model induced by CTX injection into the right tibialis anterior muscle. **a**–**c** Staining for acid phosphatase revealed a significant decrease in the lysosomal activity in mice that had been treated with the whole ADSC secretome. **d**–**g** Quantification of the cross-sectional area (CSA) demonstrated that the skeletal muscle regeneration is significantly enhanced by whole secretome, with a greater effect with the EV fraction. Scale bar: 100 μm. **h**–**k** Immunohistochemical staining and quantification of satellite cell myogenic progression showed an increase in the number of quiescent (Pax7^+^/MyoD^−^) satellite cells following whole secretome treatment. Both the whole secretome and the EV fraction decrease the number of activated (Pax7^+^/MyoD^+^) satellite cells, with a decrease in the number of differentiating satellite cells (Pax7^−^/MyoD^+^) only observed following treatment with the EV fraction (red arrow: quiescent satellite cell, yellow arrow: differentiating satellite cell, white arrow: activated satellite cell. Scale bar: 20 μm. **l**–**o** Immunohistochemical stainings for CD68 revealed that both fractions of ADSC secretome decreased the number of infiltrating macrophages. Scale bar: 50 μm. **p**–**s** Immunohistochemical analysis of CD31 within the muscle tissue showed that only the total secretome had an angiogenic effect. Scale bar: 100 μm. *p* < 0.05 (*), *p* < 0.01 (**) or *p* < 0.001 (***). Three separate batches of ADSC secretome and isolated EV fraction were tested

An acute CTX-induced muscle injury model was used to assess the impact of a systemic administration of either the whole ADSC secretome or the EV fraction on tissue regeneration. The staining for acid phosphatase (a marker for lysosomal activity) was carried out to quantify the level of muscle degeneration 5 days after the CTX injection. We found a significant decrease in the lysosomal activity in mice that had been treated with the whole ADSC secretome (Fig. 3a–c) indicating an increase in regeneration.

In order to comparatively quantify the effects of the total ADSC secretome and the EV fraction on muscle regeneration, we quantified the cross-sectional area (CSA) of newly formed muscle fibres identified by the expression of embryonic myosin heavy-chain (eMHC). We found that mice treated with the total ADSC secretome had significantly larger newly formed fibres compared to PBS-treated animals (Fig. 3d–g). Notably, the EV fraction displayed a much greater effect than the whole secretome (Fig. 3g).

To further dissect the cellular mechanisms underpinning the muscle regeneration, we quantified the myogenic cell populations in the damaged tissue. Our results showed a significant increase in the number of quiescent satellite cells (Pax7^+^/MyoD^−^) in the damaged tissue of total ADSC secretome-treated mice and a slight non-significant increase after the EV treatment (Fig. 3h, i). Analysis of differentiated satellite cells (Pax7^−^/MyoD^+^) revealed a significant decrease of Pax7^−^/MyoD^+^ cells in mice treated with the EV fraction whereas no difference was observed between total secretome-treated animals and the controls (Fig. 3j, k). In contrast, both fractions decreased the number of infiltrating macrophages (Fig. 3l–o) with the EV fraction having a significantly stronger effect. An additional analysis of angiogenesis showed a significant increase of CD31^+^ cells within damaged areas compared to the controls. Notably, the EV fraction was identified to have no pro-angiogenic effect (Fig. 3p–s).

### Characterisation of the soluble proteins and the ADSC EV protein cargo

**Fig. 4:**
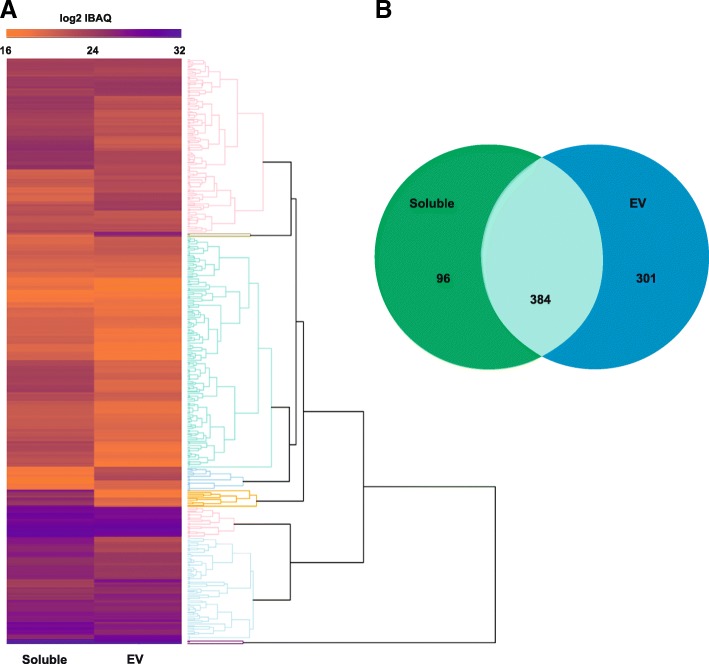
LC–MS analysis of total ADSC secretome vs. EV fraction revealed exclusively and mutually present proteins in both fractions. **a** Heat map of the proteins enriched within either the soluble or EV fractions of the ADSC secretome. **b** A total number of 781 proteins were identified from LC–MS analysis within the ADSC secretome. 301 were exclusively identified in the EV fraction, 96 in the soluble fraction and 384 are identified in both samples

LC–MS profiling with subsequent bioinformatic analysis showed a great overlap (49%) between the secreted proteins and the EV fractions with 384 of 781 proteins being mutually exclusive (Fig. 4a, b). Three hundred one proteins (38.5%) were exclusively found in EVs, and 96 (12.3%) only in the soluble fraction (Fig. 4b). This analysis also identified many exosome-specific proteins within the EV fraction (Additional file 3: Table S1). Analysis of clustering protein abundances identified a distinctively different profile between the proteins enriched in the soluble fraction compared to the EV fraction (Fig. 4a). Within the total ADSC secretome, several proteins involved in RNA metabolism have been identified using GO Enrichment Analysis (Additional file 3: Table S2). Surprisingly, no cellular processes were found to be significantly enriched within the soluble fraction, and many processes within the EV fraction were involved in protein complex disassembly and associated with the membrane (Additional file 3: Table S3).

### ADSC-derived EVs contain miRNA targeting processes involved in regeneration and regulation of inflammation

EVs were isolated from total secretome of three independent ADSC culture and the miRNA content of ADSC EV was analysed using a GeneChip® miRNA 4.0 array with the Affymetrix Expression Console™ software. We were able to detect a wide range of miRNA previously associated with regulation of regeneration and inflammation including the let 7 family [51] and miR145 [52] as well miRNAs known to target angiogenesis-related pathways (miR23a [53]).

**Fig. 5:**
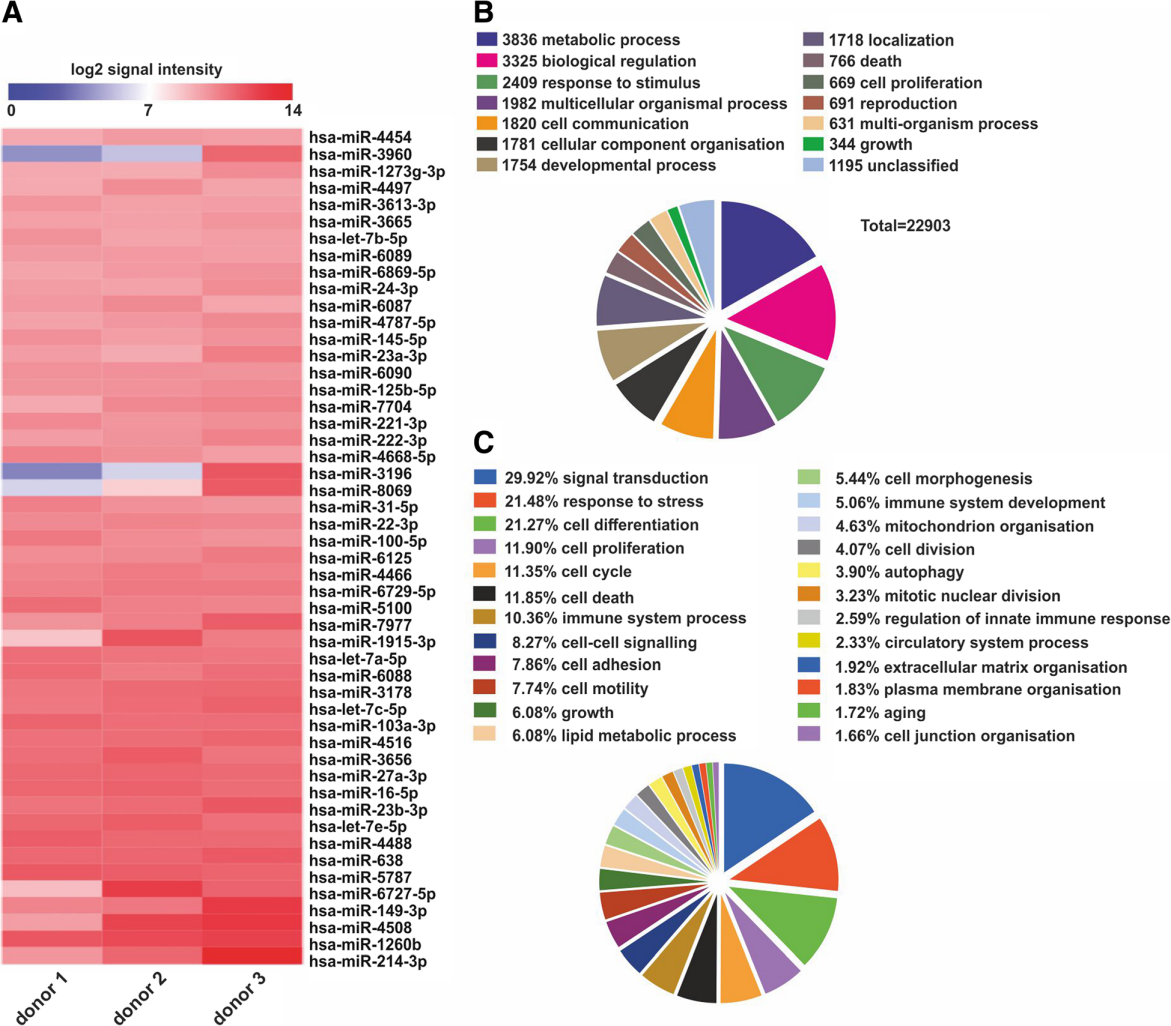
Analysis of the miRNA profile of ADSC secretome. Top 50 miRNA species hits within the ADSC secretome as determined by miRNA array analysis. **a** Heat map showing top 50 miRNA species. **b**–**c** Database mining and functional analysis mapping the biological processes of the 22,903 mRNA target hits from the miRNA array

Top 50 miRNAs were plotted in a heat map to show the intensity variability between the samples (Fig. 5a).

Validated miRNA targets were amalgamated using miRWalk2.0 software, and their biological processes were categorised using GL Slim classification with WebGestalt software. This analysis identified a total of 22,903 validated targets of the miRNAs, with over half of the mRNA targets being linked to just four biological processes: metabolic processes, biological regulation, response to stimulus and multicellular organismal process (Fig. 5b). Mapping of the individual validated targets to biological processes found a large proportion (29.92%) to be linked to signal transduction (Fig. 5c).

### ADSC and AFSC secretomes have a distinct molecular profile despite similar biological activity in muscle regeneration

**Fig. 6:**
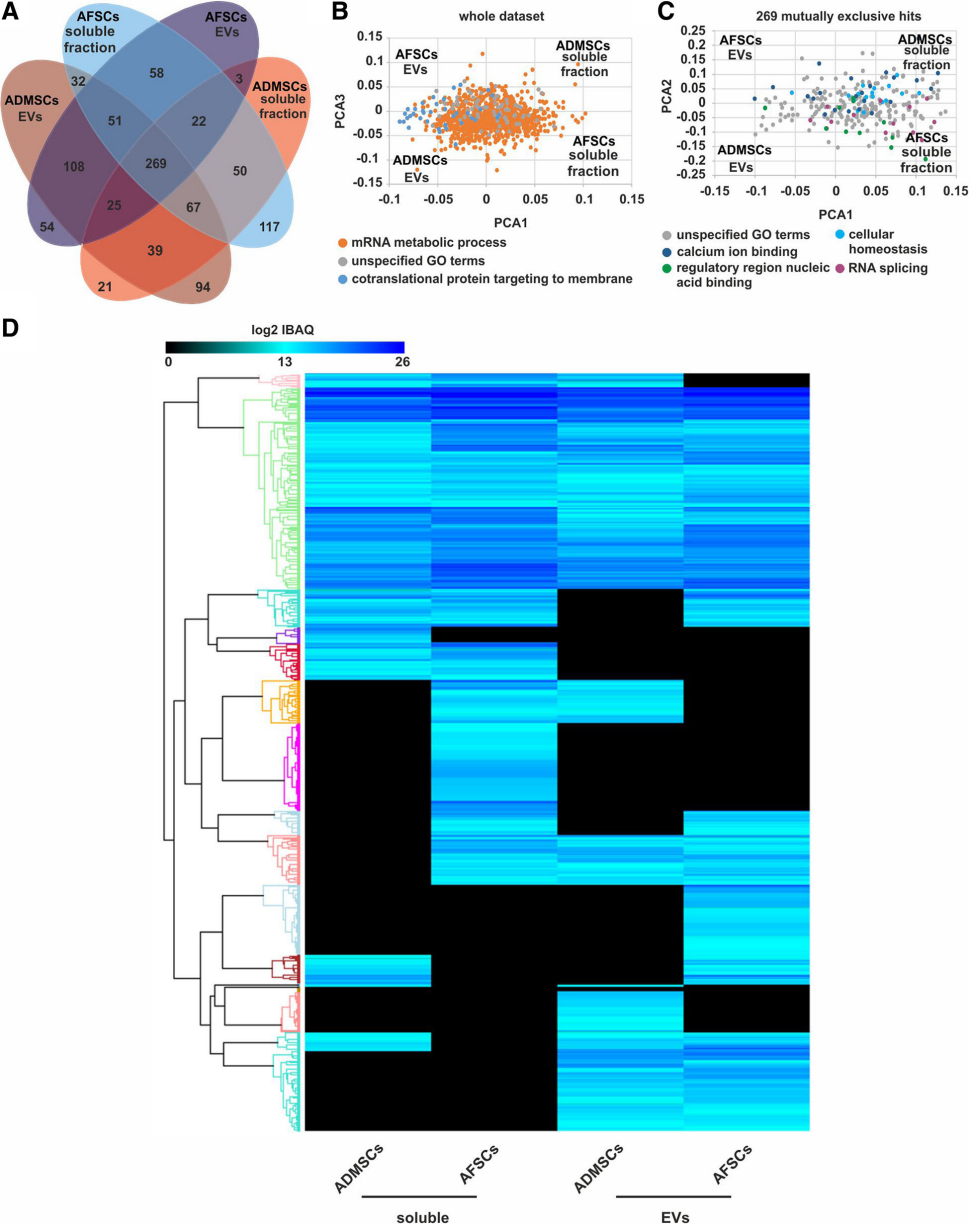
Comparative analysis of the proteins in soluble and EV fractions within the secretome of ADSCs and AFSCs revealed distinct profiles***.*** **a** Numerical Venn diagram of identified hits. **b** PCA analysis using the entire dataset and **c** 269 proteins identified in all samples, highlighted in both **b** and **c** the most represented GO terms categories based on Benjamini-Hochberg FDR values. **d** Heat map reporting all identified hits and considering log2-transformed iBAQ quantification values

Finally, we have taken a bioinformatic approach to compare molecular components of the ADSC secretome with the secretome of AFSCs that also promotes muscle regeneration [38]. Using this approach, we found 108 mutually exclusive protein hits in the EV fractions of both secretomes and 50 proteins present in both soluble fractions (Fig. 6a).

Interestingly, 269 proteins were mutually expressed in both fractions of ADSCs and AFSCs (Fig. 6a). Functional enrichment analysis of all fractions revealed strong enrichment relating to the immune system and exocytosis (Additional file 3: Table S4).

Principal component analysis (PCA) of all samples revealed that EV fractions of both cell types mainly contain proteins linked to ‘cotranslational protein targeting to the membrane’ (Fig. 6b, Additional file 3: Table S5) whereas proteins mutually present in the soluble fractions were classified as ‘proteins involved in mRNA metabolic processes’ (Fig. 6b). In contrast to the functional enrichment analysis carried out for the soluble fractions, no considerable functional enrichment was found (Additional file 3: Table S6).

**Fig. 7:**
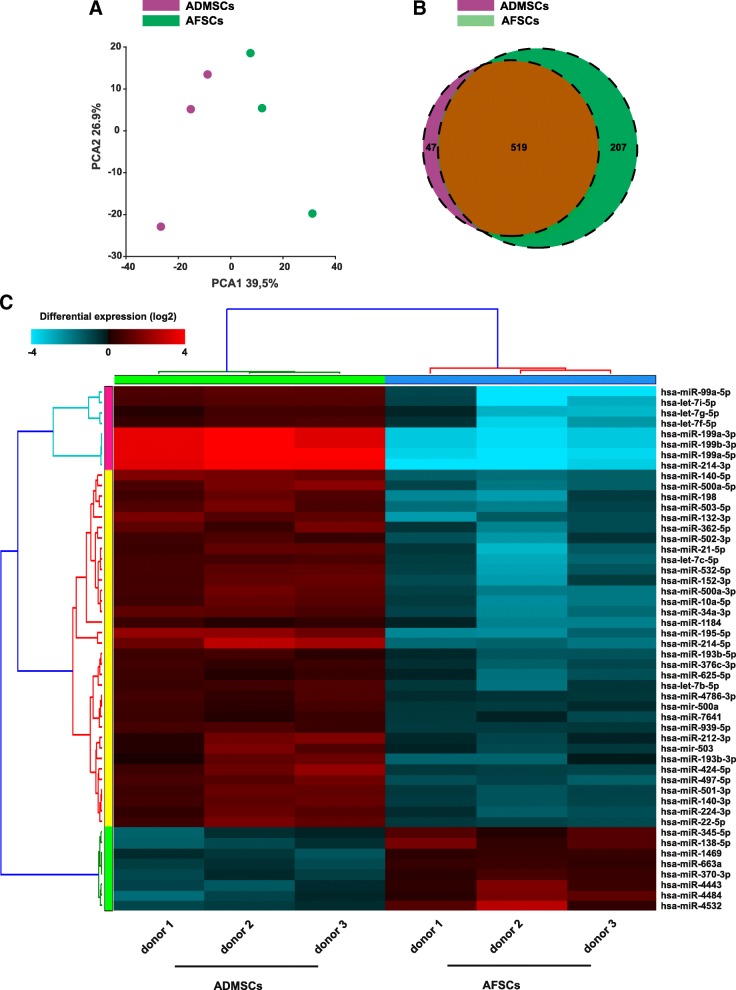
Comparative analysis of the miRNA profiles of ADSCs versus AFSCs demonstrated a distinct profile with mutually present and exclusive miRNAs***.*** **a** 2D PCA plot shows two distinct clusters along the PC1 axis that correspond to the ADSCs (red) and AFSCs (green). **b** Weighted Venn diagram. The graph consists of miRNAs expressed in ADSCs (red) and in AFSCs (green). 47 miRNAs were found to be exclusively present in the ADSC samples whereas 207 miRNA were exclusive for AFSCs. 519 miRNA were mutually found in both cell types. **c** Hierarchical clustering heat map of the top 50 regulated miRNAs in multiple comparisons. MicroRNAs are displayed in the rows and samples in the columns. The expression levels are indicated via the colour coding. The shades of blue and red refer to the absolute expression levels; the brighter the blue, the lower the expression level, and the brighter the red, the higher the expression level. The dendrograms are based on cosine column/row clustering

PCA of the miRNA cargo of EVs from ADMSCs and AFSCs revealed that the miRNA profiles are distinct (Fig. 7a). However, 519 mutually exclusive miRNAs and only 47 miRNAs exclusively present in ADMSC EVs (Fig. 7b) have been identified. Notably, mutually present miRNAs included the anti-inflammatory let7 family (Fig. 7c).

## Discussion

MSCs are a clinically relevant adult stem cell population with the potential to positively modulate muscle regeneration in acute and chronic muscle injury [6, 7, 54]. Conditioned media from stem cells and their EVs are typically collected from basal medium, or complete serum-based media [8, 19, 34, 55].

The utilisation of PBS as the carrier for EVs and soluble components of the secretome reduces the risk of transmission of xenogenic substances. Thus, the secreted products would have the potential to be clinically compliant (saline solution). The hypoxic (cell pelleted) environment also induces a cellular stress that would enhance the secretion of molecules that may aid regeneration [56]. Moreover, in a model of muscle degeneration, EVs isolated from MSCs cultivated under hypoxic conditions have shown to be more efficient in promoting regeneration than EV from normoxic MSCs [19].

Our characterisation of the total ADSC secretome, the isolated EV fraction and the EV-depleted (soluble) fraction showed that each contained a distinct spectrum of proteins (Fig. 1a, b). Although the presence of mRNA molecules has been reported in secretomes of MSCs in other groups [3], we did not detect RNA molecules larger than ~ 31 nucleotides (Fig. 1c). Notably, the lack of mRNA molecules in the ADSC secretome is consistent with our results obtained with AFSC secretome and might be a consequence of the collection protocol [38]. Using TEM and NTA, we show that the EVs had an average size of 57 nm, fitting well with the reported characteristics for exosomes (30–100 nm) [57]. Our mass spectrometry profile allowed us to identify many exosome-specific proteins within our EV fraction, such as CD63, HSPs and LAMP2 (Additional file 3: Table S1) but, however, failed to record evidence of other commonly used exosome markers CD9 and CD81 [33, 58, 59]. Further validation of these missing proteins via western blot also provided a negative result (data not shown). Their absence in our EV fraction could possibly be explained by a low concentration of the proteins or via an interference of the EV structure and composition following the denaturation steps we carried out for both western blot and LC–MS [60].

Using PHK67-labelled ADSC EVs and IMR-90 cells visualised with phalloidin, we showed an intracellular co-localisation of PKH67 and phalloidin signal suggesting that our ADSC EVs were able to be taken up by host cells in vitro (Fig. 1h, white arrow). MSC EVs are thought to utilise the same mechanism as their parent cell for ‘homing’ to a site of injury by using surface receptors and adhesion molecules preserved from the MSC cytomembrane during EV formation [61].

We show that the total ADSC secretome positively affects proliferation of muscle cells and provides protection against stress-induced cellular senescence. However, in the present study, no significant effects on migration speed have been observed (Fig. 2a, d, f).

In the present study, we assessed the influence of ADSC-secretome and ADSC-derived EVs on H_2_O_2_-induced senescence of IMR-90 cells, a widely used model in cell ageing research [62–64]. Notably, only the total secretomes but not the EV fractions were able to reduce the number of senescent cells (Fig. 2f) suggesting that soluble factors rather than EV cargo is responsible for this effect.

In contrast, total ADSC failed to impact nuclear translocation of the NF-κB assay whereas the EV fraction did (Fig. 2g, h), implying that the anti-inflammatory effects of the ADSC secretome are mainly mediated by the EV fraction. This is in line with recent reports showing strong anti-inflammatory properties of MSC-derived EVs in different pathological scenarios [19, 65, 66]. Carrying out similar experiments with a control fibroblast secretome saw no effect on the same in vitro assays, suggesting that the effects we observed are due to the regenerative nature of the ADSC source, rather than a concentration of secreted products.

The study of Lo Sicco et al. [19] is of particular significance to this work, since they found that hypoxic conditions supported the development of a potent regenerative EV fraction. However, it seems that MSCs are extremely labile in terms of the secretome repertoire. Indeed, although a number of groups have shown that the EV fraction of MSC secretomes support muscle regeneration [19, 67], the identity of the putative effective agents differs greatly. For example, Nakamura suggested that the angiogenesis supporting property lay with non-EV VEGF and that the EV encapsulated miR494 promoted myogenesis. Although we see both properties, our EV free fraction lacked VEGF, as well as other angiogenic proteins such as SPRED1, VECAM1 and IGF1, and the EV fraction contained low levels of miR494. This suggests that there are multiple routes to promote key features of muscle regeneration and demonstrates the potential diversity that may result from differential secretome generation protocols. In order to identify the soluble and EV-associated protein components potentially contributing to the effect of the ADSC secretome, we performed an LC–MS profiling with subsequent bioinformatic analysis (Figs. 4 and 6). We detected many proteins in the soluble fraction that have previously been described in the MSC secretome, including Heat Shock Proteins (HSPs) (HSP60, HSP90, HSP105). HSPs are produced by cells in response to stressful conditions, such as heat, wound healing or inflammation [68–70] Notably, HSPs have also been shown to enhance regeneration in MSC therapies [58, 71]. Our analysis also revealed the presence of superoxide dismutase (SOD2) that is known to mediate resistance to oxidative stress [72].

Paracrine factors released by MSCs are known to positively influence the levels of angiogenesis [25, 73]. In particular, HGF, bFGF, IGF-1, and VEGF have been identified as paracrine factors responsible for the pro-angiogenic effects of MSCs [74]. Due to the strong pro-angiogenic effects of the total ADSC secretome but not the EV fraction, we expected that the soluble protein fraction would contain all or at least some of these. To our surprise, our LC–MS analysis revealed a lack of these growth factors within the soluble fraction. This may be explained by the concentrations of these proteins being below the sensitivity of the analysis but still exerting biological activity [75].

miRNAs transported within EVs have previously been shown to be able to regulate gene expression in distant tissues [76]. Moreover, it has been reported that miRNA can be released into blood plasma via packaging within EVs [34, 76, 77], bound to RNA-binding proteins such as Argonaute (Ago) or lipoprotein complexes including high-density lipoprotein (HDL) [78, 79]. Notably, although our LC–MS analysis did not detect Ago, HDL is present.

Our observation that ADSC secrete a large number of miRNA species advocates the enormous potential of its secretome in modifying the surrounding microenvironment, with each miRNA possibly effecting hundreds of different mRNA targets, and therefore being able to affect numerous signalling pathways [80].

Analysis of the miRNAs within the EV fraction of the ADSC secretome revealed a presence of many well-characterised anti-inflammatory miRNAs, including the miR-let7 family (let7a, b, c and e). miR-let7c in particular has been demonstrated to be expressed at a higher level in anti-inflammatory M2 macrophages [81] and to play an important role in reducing fibrosis, a key factor in chronic inflammation [82]. In contrast, let 7b, another let7 family member identified in the top 50 miRNA species, targets the pro-inflammatory signalling axis of TLR4 [83]. Notably, let7b within MSC EVs has previously been reported to be able to modify macrophage polarisation towards the anti-inflammatory M2 phenotype [51]. We were also able to detect miRNA targeting other downstream molecules in the Toll-like receptor signalling pathway including miR-24 (targets: MyD88 and NF-κB [84]), miR-125b (targets: IL-6, TNF-α [85, 86]) and miR-16 (target: IKK-α [87]).

Overall, we found that a large proportion of the validated miRNA targets play a role in the regulation of immune systems processes, immunological development and in the regulation of the innate immune response. Moreover, two of the top 50 miRNAs within the ADSC EVs, miR-23a and miR-23b, are known to be strongly pro-angiogenic [53].

A brief comparison of the miRNA content within the ADSC secretome and those described in literature [19, 38, 88] identifies that the pre-conditioning protocol utilised to generate a secretome may be the key factor governing the miRNA cargo rather than the cell type (Additional file 3: Table S7).

In order to compare the active compounds within the ADSC secretome to another secretome recently described to promote muscle regeneration (AFSCs [38]), we performed an in-depth bioinformatic analysis of the respective soluble and EV fractions (Fig. 6), identifying numerous mutually exclusive proteins in each of the different fractions. A PCA and cluster analysis revealed that secretomes of both stem cell populations have a distinct protein profile despite similar biological activity. GO enrichment analysis of the whole dataset showed that all clusters were mostly associated with ‘mRNA metabolic processes’ and ‘cotranslational protein targeting to membrane’ whereas that the mutually exclusive hits were mostly classified as ‘unspecified GO terms’.

PCA showed distinct clustering for both stem cell types further unlinking the differences in the miRNA cargo (Fig. 6b–d). Notably, several mutually exclusive miRNAs were anti-inflammatory including let7b, miR-22 [89], miR-199a (target: NF-κB pathway) [90] and the key switch in inflammatory response miR-21 [91]. Moreover, we were also able to detect the pro-angiogenic miR-132 [92]. Interestingly, although present in both fractions, members of the let7 family showed highly differential expression level. This comparative analysis of the protein and miRNA components of both stem cell types reveals distinct profiles despite similar biological effects in muscle regeneration.

## Conclusions

Overall, our data suggest that soluble and EV-associated factors promote muscle regeneration both in vitro and in vivo in a highly synergistic manner. This is supported by our findings showing that both fractions affect different aspects of tissue regeneration after muscle injury. Moreover, our comparative analysis of ADSC and AFSC secretomes indicates that different molecular factors might mediate similar beneficial biological outcomes.

## Additional files

Additional file 1: **Figure S1.** ADSCs retain their multipotency following secretome generation. (A–B) FACS analysis before and after generation of the secretome revealed that there was no change in expression pattern of ADSCs after 24 h in PBS. (C) Surviving cells readily differentiated into Alizarin Red-positive osteogenic cells, (D) Oil Red O-positive adipocytes, and (E) Alcian blue-positive chondrogenic cells. Scale bar: 100 μm. (JPG 339 kb)

Additional file 2: **Figure S2.** (A) The secretome generated from fibroblast cells under identical conditions to ADSC contains a significantly lower amount of total protein. (B-C) Characterising the fibroblast EVs using NTA found a significantly lower concentration per mL, with a similar size range to ADSC EVs (< 500 nm). (D-F) Investigating the functional characteristics of the fibroblast secretome found no significant increase in cellular proliferation compared to PBS control (D). There was no change from control levels of C2C12 cell fusion with treatment with fibroblast secretome (E). Fibroblast secretome demonstrated a slight non-significant increase in percentage gap closure on the in vitro wound assay (F). *p* < 0.05 (*), *p* < 0.01 (**) or *p* < 0.001 (***). Three batches of fibroblast secretome were generated and tested. (JPG 243 kb)

Additional file 3: **Table S1.** Exosome proteins enriched within the EV fraction of the whole ADSC secretome. **Table S2.** Soluble and EV GO-MF terms enrichment. Table S3 EV fraction GO-MF terms enrichment. **Table S4.** Top 20 significantly enriched GO BP terms secretome-wide. **Table S5.** Top 20 significantly enriched GO BP terms in ADSC and AFS EV fractions. **Table S6.** Top significantly enriched GO BP terms in ADSC and AFS soluble fractions. **Table S7.** Comparison of miRNA cargo between different ADSC and AFS cell types and secretome pre-conditioning methods. (DOCX 51 kb)

## Abbreviations

ADSC: Adipose-derived mesenchymal stem cells
AFSC: Amniotic fluid stem cells
CNS: Central nervous system
CTX: Cardiotoxin
eMHC: Embryonic myosin heavy-chain
EV: Extracellular vesicles
FBS: Fetal bovine serum
HGF: Hepatocyte growth factor
IGF-1: Insulin-like growth factor-1
LC–MS: Liquid chromatography mass spectrometry
MSC: Mesenchymal stem cells
MV: Microvesicles
NF-κB: Nuclear factor kappa-light-chain-enhancer of activated B cells
NTA: Nanoparticle` tracking analysis
PBS: Phosphate-buffered saline
SDS-PAGE: Sodium dodecyl sulfate polyacrylamide gel electrophoresis
TA: Tibialis anterior
TEM: Transmission electron microscopy
TNF-α: Tumour necrosis factor alpha
VEGF: Vascular endothelial growth factor
