# Supplementary figures and images for: Secretome of adipose-derived mesenchymal stem cells promotes skeletal muscle regeneration through synergistic action of extracellular vesicle cargo and soluble proteins
Source: Stem Cell Res Ther. 2019 Apr 5;10:116. doi: 10.1186/s13287-019-1213-1 (PMC6451311; doi:10.1186/s13287-019-1213-1)

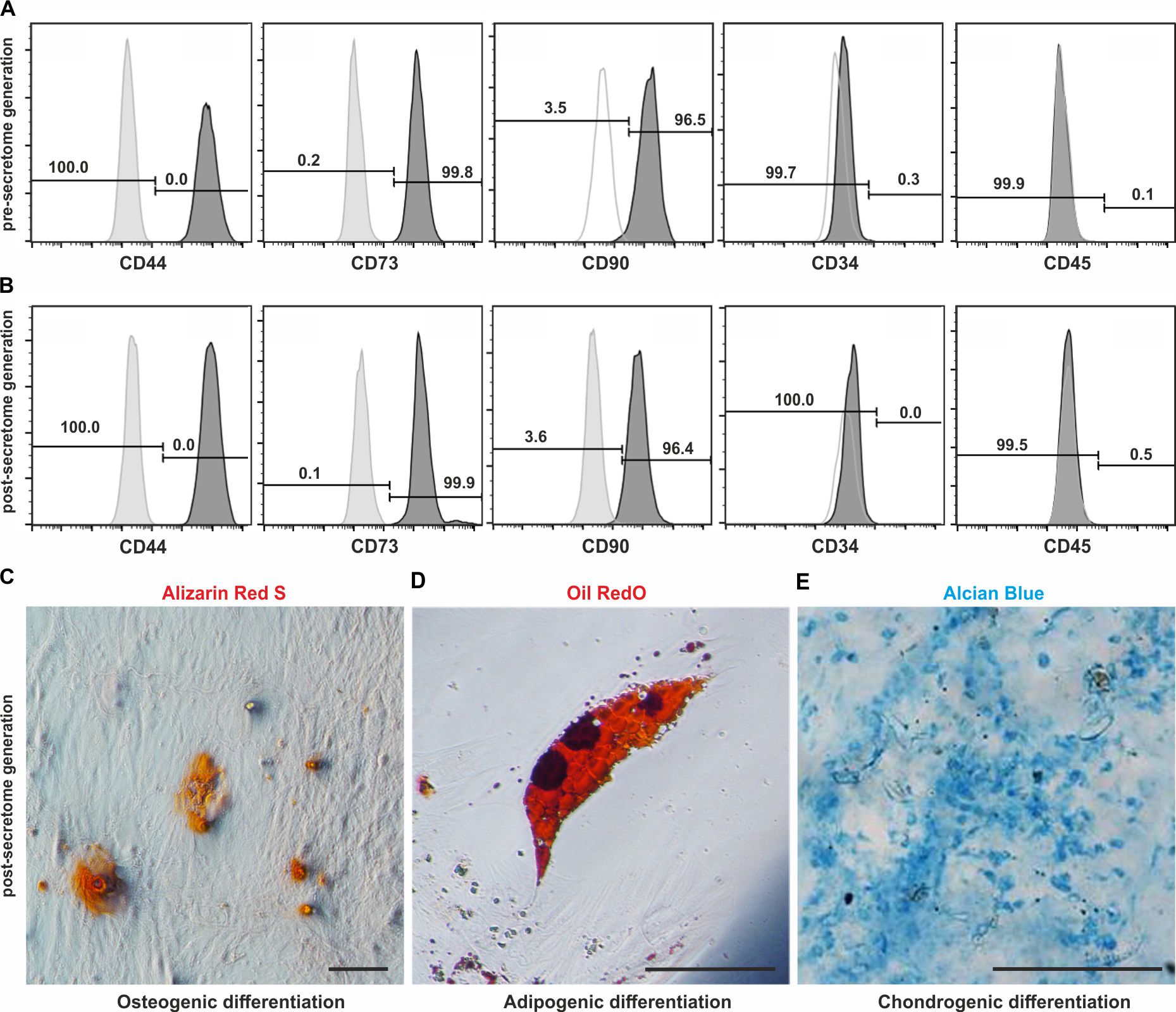

Supplement: Supplementary file 1 — Figure S1. ADSCs retain their multipotency following secretome generation. (A–B) FACS analysis before and after generation of the secretome revealed that there was no change in expression pattern of ADSCs after 24 h in PBS. (C) Surviving cells readily differentiated into Alizarin Red-positive osteogenic cells, (D) Oil Red O-positive adipocytes, and (E) Alcian blue-positive chondrogenic cells. Scale bar: 100 μm. (JPG 339 kb) [file 13287_2019_1213_MOESM1_ESM.jpg]

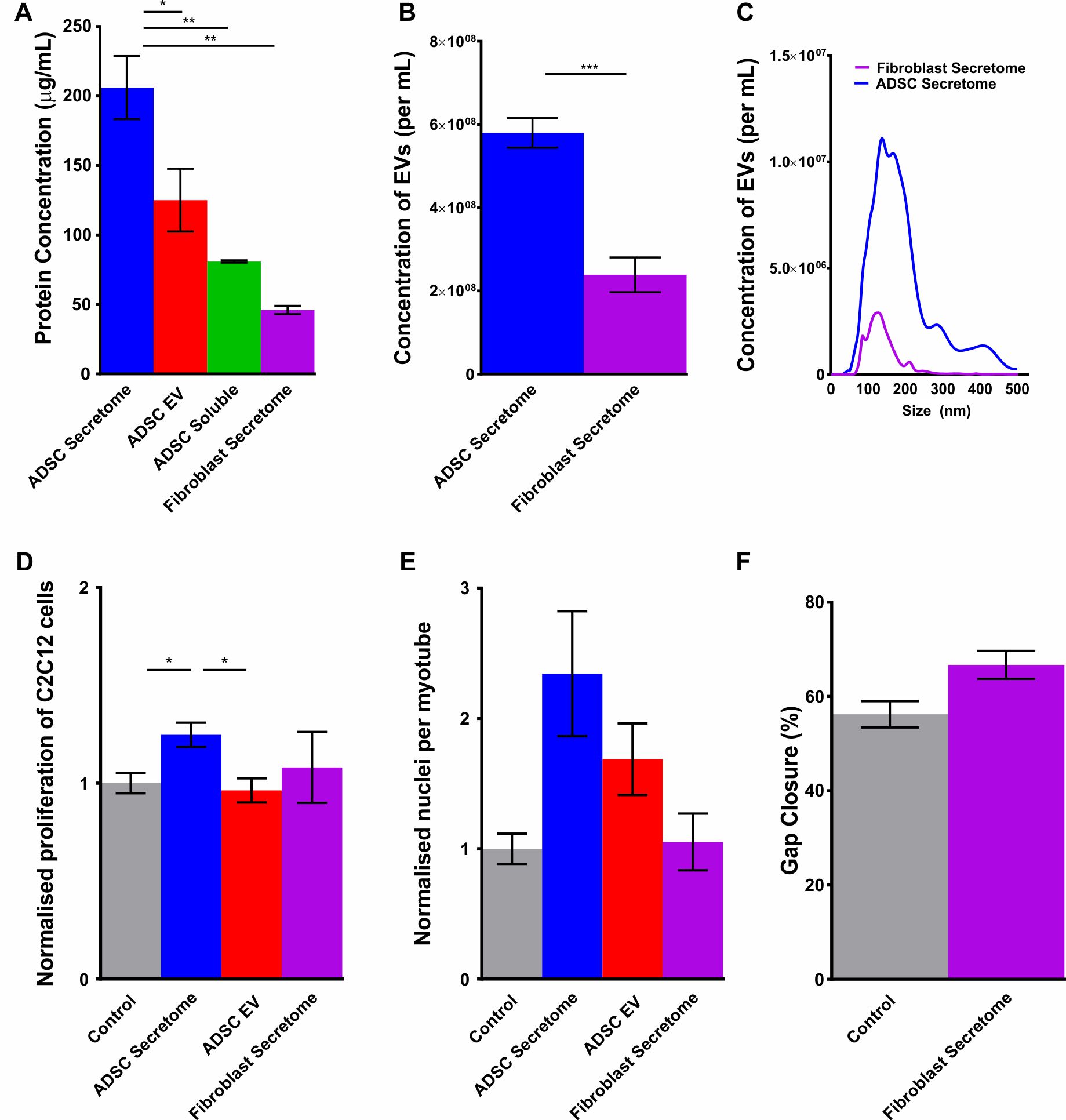

Supplement: Supplementary file 2 — Figure S2. (A) The secretome generated from fibroblast cells under identical conditions to ADSC contains a significantly lower amount of total protein. (B-C) Characterising the fibroblast EVs using NTA found a significantly lower concentration per mL, with a similar size range to ADSC EVs (< 500 nm). (D-F) Investigating the functional characteristics of the fibroblast secretome found no significant increase in cellular proliferation compared to PBS control (D). There was no change from control levels of C2C12 cell fusion with treatment with fibroblast secretome (E). Fibroblast secretome demonstrated a slight non-significant increase in percentage gap closure on the in vitro wound assay (F). p < 0.05 (*), p < 0.01 (**) or p < 0.001 (***). Three batches of fibroblast secretome were generated and tested. (JPG 243 kb) [file 13287_2019_1213_MOESM2_ESM.jpg]
