# Supplementary material for: Secretome of adipose-derived mesenchymal stem cells promotes skeletal muscle regeneration through synergistic action of extracellular vesicle cargo and soluble proteins
Source: Stem Cell Res Ther. 2019 Apr 5;10:116. doi: 10.1186/s13287-019-1213-1 (PMC6451311; doi:10.1186/s13287-019-1213-1)
Supplement: Supplementary file 3 — Table S1. Exosome proteins enriched within the EV fraction of the whole ADSC secretome. Table S2. Soluble and EV GO-MF terms enrichment. Table S3 EV fraction GO-MF terms enrichment. Table S4. Top 20 significantly enriched GO BP terms secretome-wide. Table S5. Top 20 significantly enriched GO BP terms in ADSC and AFS EV fractions. Table S6. Top significantly enriched GO BP terms in ADSC and AFS soluble fractions. Table S7. Comparison of miRNA cargo between different ADSC and AFS cell types and secretome pre-conditioning methods. (DOCX 51 kb) [file 13287_2019_1213_MOESM3_ESM.docx]

Additional file 3

Table S1 Exosome proteins enriched within the EV fraction of the whole ADSC secretome.

| Pathway | Protein | Reference |
| --- | --- | --- |
| Tetraspanins | CD63 | (Cheng, Zhang, Wu, Cui, & Xu, 2017; Koniusz et al., 2016) |
| Heat shock proteins: | HSP 70,  HSP 90,  HSP 105,  beta 1, | (Cheng et al., 2017; Koniusz et al., 2016) |
| Lysosomal proteins: | LAMP2 | (Cheng et al., 2017; Koniusz et al., 2016) |
| Fusion proteins: | Annexin | (Cheng et al., 2017) |
| GTPases | Elongation factor 1-alpha 1: EEF1A1,  Eukaryotic Translocation Elongation Factor 2: EEF2 | (Sarvar, Shamsasenjan, & Akbarzadehlaleh, 2016) |
| Metabolic enzymes: | Glyceraldehyde 3-phosphate dehydrogenase: GAPDH,  Lactate Dehydrogenase A: LDHA,  Phosphoglycerate Kinase 1: PGK1 | (Sarvar et al., 2016) |
| MSC markers: | CD44 | (Sarvar et al., 2016) |

Table S2 Soluble and EV GO-MF terms enrichment

| Category value | Selection | Category | Enrichment factor | P value | Benj. Hoch. FDR |
| --- | --- | --- | --- | --- | --- |
| Aminoacyl-tRNA biosynthesis | 13 | 13 | 29,538 | 1,94E-24 | 9,24E-20 |
| Cytoskeleton | 43 | 78 | 4,3506 | 2,99E-25 | 1,28E-19 |
| small molecule metabolic process | 172 | 96 | 2 | 5,23E-26 | 1,50E-19 |
| metabolic process | 172 | 253 | 1,4031 | 2,35E-25 | 3,35E-19 |
| nucleic acid metabolic process | 85 | 84 | 2,958 | 7,27E-24 | 1,73E-18 |
| primary metabolic process | 172 | 227 | 1,4359 | 1,63E-21 | 2,91E-16 |
| regulation of catalytic activity | 33 | 59 | 5,5223 | 3,02E-21 | 5,08E-16 |
| ncRNA metabolic process | 13 | 17 | 22,588 | 4,61E-21 | 5,73E-16 |
| proteolysis | 26 | 30 | 9,8462 | 4,84E-21 | 5,77E-16 |
| ncRNA metabolic process | 13 | 17 | 22,588 | 4,61E-21 | 5,99E-16 |
| tRNA aminoacylation for protein translation | 17 | 13 | 22,588 | 4,61E-21 | 6,28E-16 |
| ribonucleoprotein complex | 64 | 44 | 4,5 | 9,05E-21 | 1,95E-15 |
| proteolysis | 27 | 30 | 9,4815 | 1,82E-20 | 2,08E-15 |
| RNA metabolic process | 85 | 73 | 2,9705 | 4,40E-20 | 4,84E-15 |
| tRNA metabolic process | 17 | 14 | 20,975 | 6,39E-20 | 6,76E-15 |
| cellular metabolic process | 172 | 229 | 1,4136 | 7,21E-20 | 7,37E-15 |
| cellular macromolecule biosynthetic process | 85 | 59 | 3,216 | 3,66E-19 | 3,49E-14 |
| actin cytoskeleton | 25 | 36 | 8,5333 | 2,57E-19 | 3,68E-14 |
| oxoacid metabolic process | 172 | 47 | 2,2326 | 7,35E-19 | 6,18E-14 |
| organic acid metabolic process | 172 | 47 | 2,2326 | 7,35E-19 | 6,37E-14 |
| cellular ketone metabolic process | 172 | 47 | 2,2326 | 7,35E-19 | 6,57E-14 |
| protein folding | 20 | 37 | 9,3405 | 8,79E-19 | 7,18E-14 |
| regulation of gene expression | 85 | 89 | 2,6395 | 1,21E-18 | 9,62E-14 |
| actin cytoskeleton | 43 | 36 | 5,9535 | 6,33E-18 | 5,44E-13 |

Table S3 EV fraction GO-MF terms enrichment

| Category value | Selection | Category | Enrichment factor | P value | Benj. Hoch. FDR |
| --- | --- | --- | --- | --- | --- |
| Ribosome | 25 | 25 | 12,04 | 4,69E-37 | 6,04E-32 |
| translational termination | 25 | 25 | 12,04 | 4,69E-37 | 1,02E-30 |
| viral transcription | 25 | 26 | 11,577 | 1,22E-35 | 3,81E-30 |
| viral reproductive process | 25 | 26 | 11,577 | 1,22E-35 | 4,44E-30 |
| viral infectious cycle | 25 | 26 | 11,577 | 1,22E-35 | 5,33E-30 |
| translational elongation | 25 | 26 | 11,577 | 1,22E-35 | 6,66E-30 |
| protein complex disassembly | 25 | 26 | 11,577 | 1,22E-35 | 8,88E-30 |
| cellular protein complex disassembly | 25 | 26 | 11,577 | 1,22E-35 | 1,33E-29 |
| SRP-dependent cotranslational protein targeting to membrane | 25 | 27 | 11,148 | 1,65E-34 | 3,27E-29 |
| protein targeting to ER | 25 | 27 | 11,148 | 1,65E-34 | 3,60E-29 |
| establishment of protein localization in endoplasmic reticulum | 25 | 27 | 11,148 | 1,65E-34 | 4,00E-29 |
| cotranslational protein targeting to membrane | 25 | 27 | 11,148 | 1,65E-34 | 4,50E-29 |
| nuclear-transcribed mRNA catabolic process | 25 | 28 | 10,75 | 1,54E-33 | 2,40E-28 |
| macromolecular complex disassembly | 25 | 28 | 10,75 | 1,54E-33 | 2,58E-28 |
| cellular macromolecular complex disassembly | 25 | 28 | 10,75 | 1,54E-33 | 2,80E-28 |

Table S4 Top 20 significantly enriched GO BP terms secretome-wide

| p-value | GO term ID | GO term | Semantic class |
| --- | --- | --- | --- |
| 3.1E-24 | GO:0006457 | protein folding | protein metabolism - folding |
| 2.46E-20 | GO:0045055 | regulated exocytosis | transport - exocytosis |
| 2.8E-20 | GO:0016192 | vesicle-mediated transport | transport - intracellular - vesicle |
| 2.89E-20 | GO:0006887 | exocytosis | transport - exocytosis |
| 1.98E-19 | GO:0036230 | granulocyte activation | immune system - leukocytes |
| 4.77E-19 | GO:0002283 | neutrophil activation involved in immune response | immune system - leukocytes |
| 4.77E-19 | GO:0043312 | neutrophil degranulation | immune system - leukocytes |
| 6.53E-19 | GO:0002275 | myeloid cell activation involved in immune response | immune system - gene expression |
| 9.53E-19 | GO:0042119 | neutrophil activation | immune system - leukocytes |
| 1.13E-18 | GO:0002446 | neutrophil mediated immunity | immune system - leukocytes |
| 5.06E-18 | GO:0043299 | leukocyte degranulation | immune system - leukocytes |
| 8.22E-18 | GO:0002444 | myeloid leukocyte mediated immunity | immune system - leukocytes |
| 2.16E-17 | GO:0051179 | localization | protein metabolism - localisation |
| 2.35E-17 | GO:0051234 | establishment of localization | protein metabolism - localisation |
| 4.26E-17 | GO:0002274 | myeloid leukocyte activation | immune system - differentiation |
| 6.3E-17 | GO:0032940 | secretion by cell | transport - exocytosis |
| 2.23E-16 | GO:0002366 | leukocyte activation involved in immune response | immune system - leukocytes |
| 2.94E-16 | GO:0002263 | cell activation involved in immune response | immune system |
| 9.19E-16 | GO:0046903 | secretion | transport - exocytosis |
| 5.85E-15 | GO:0001775 | cell activation | general |

Table S5 Top 20 significantly enriched GO BP terms in ADSC and AFS EV fractions

| p-value | GO term ID | GO term | Semantic class |
| --- | --- | --- | --- |
| 2.56E-32 | GO:0006614 | SRP-dependent cotranslational protein targeting to membrane | protein metabolism - localisation - membrane |
| 2.31E-31 | GO:0006613 | cotranslational protein targeting to membrane | protein metabolism - localisation - membrane |
| 4.2E-31 | GO:0045047 | protein targeting to ER | protein metabolism - localisation - ER |
| 1.32E-30 | GO:0072599 | establishment of protein localization to endoplasmic reticulum | protein metabolism - localisation - ER |
| 2.59E-30 | GO:0070972 | protein localization to endoplasmic reticulum | protein metabolism - localisation - ER |
| 9.58E-30 | GO:0016071 | mRNA metabolic process | RNA metabolism |
| 2.43E-29 | GO:0000184 | nuclear-transcribed mRNA catabolic process, nonsense-mediated decay | RNA metabolism - stability |
| 8.57E-29 | GO:0006413 | translational initiation | RNA metabolism - translation |
| 2.57E-27 | GO:0006401 | RNA catabolic process | RNA metabolism |
| 3.77E-27 | GO:0006402 | mRNA catabolic process | RNA metabolism |
| 7.47E-26 | GO:0006612 | protein targeting to membrane | protein metabolism - localisation - membrane |
| 1.84E-24 | GO:0019083 | viral transcription | physiology - virus |
| 1.53E-23 | GO:0019080 | viral gene expression | physiology - virus |
| 1.56E-23 | GO:0034655 | nucleobase-containing compound catabolic process | metabolism |
| 2.36E-22 | GO:0000956 | nuclear-transcribed mRNA catabolic process | RNA metabolism |
| 2.41E-22 | GO:0022613 | ribonucleoprotein complex biogenesis | RNA metabolism |
| 2.44E-22 | GO:0046700 | heterocycle catabolic process | metabolism |
| 2.79E-22 | GO:0044270 | cellular nitrogen compound catabolic process | metabolism |
| 5.42E-22 | GO:0019439 | aromatic compound catabolic process | metabolism |
| 6.39E-22 | GO:0006412 | translation | RNA metabolism - translation |

Table S6 Top significantly enriched GO BP terms in ADSC and AFS soluble fractions

| p-value | GO term ID | GO term | Semantic class |
| --- | --- | --- | --- |
| 0.00346 | GO:0008635 | activation of cysteine-type endopeptidase activity involved in apoptotic process by cytochrome c | cell death - enzymes |
| 0.0211 | GO:0006749 | glutathione metabolic process | metabolism |

Table S7 Comparison of miRNA cargo between different ADSC and AFS cell types and secretome pre-conditioning methods.

| **ADSC** | **AFS** | **(Wen et al., 2014)** | **(Lo Sicco et al., 2017)** | |
| --- | --- | --- | --- | --- |
| **Top Expressed** | **Top Expressed** | **Top Expressed** | **Under Expressed** | **Over Expressed** |
| miR-4454 | miR-1273g-3p | miR-652 | miR-376c | miR-855-5p |
| miR-3960 | miR-4454 | miR-29c | miR-708 | miR-872 |
| miR-1273g-3p | miR-3613-3p | miR-222 | miR-539 | miR-636 |
| miR-4497 | miR-3960 | miR-146a | miR-494 | miR-628-5p |
| miR-3613-3p | miR-3665 | miR-377 | miR-484 | miR-518f |
| miR-3665 | miR-6087 | miR-195 | miR-411 | miR-509-5p |
| miR-6089 | miR-6089 | miR-146b | miR-382 | miR-454 |
| Let-7b-5p | miR-6090 | miR-92a | miR-376a | miR-451 |
| miR-6869-5p | miR-7704 | miR-323 | miR-370 | miR-374-5p |
| miR-24-3p | miR-24-3p | miR-877 | miR-345 | miR-302c |
| miR-6087 | miR-4787-5p | miR-466c | miR-155 | miR-202a |
| miR-4787-5p | miR-4668-5p | miR-466b | miR-324-3p | miR-223 |
| miR-145-5p | miR-6869-5p | miR-196c | miR-301 | miR-214 |
| miR-23a-3p | miR-4497 | miR-664 | miR-210 | miR-199a-3p |
| miR-6090 | miR-23a-3p | miR-466d | miR-196b | miR-184 |
| miR-125b-5p | miR-8069 | let-7b | miR-194 | miR-150 |
| miR-7704 | miR-4508 | miR-685 | miR-181a | miR-146b |
| miR-221-3p | miR-6088 | miR-351 | miR-149 | miR-142-3p |
| miR-222-3p | miR-6729-5p | miR-3541 | miR-146a | miR-139-5p |
| miR-4668-5p | miR-6125 | miR-181c | miR-138 | miR-133b |
| miR-3196 | miR-3196 | miR-142-5p | miR-130b | miR-126 |
| miR-8069 | miR-4466 | miR-92b | miR-127 | miR-122 |
| miR-31-5p | miR-5787 | miR-323 | miR-100 |  |
| miR-22-3p | miR-221-3p | miR-182 | miR-99b |  |
| miR-100-5p | miR-4516 | miR-1 | miR-99a |  |
| miR-6125 | miR-1915-3p | miR-3591 | miR-34a |  |
| miR-4466 | miR-149-3p | miR-140 | miR-31 |  |
| miR-6729-5p | miR-320a | miR-708 | miR-30c |  |
| miR-5100 | miR-6727-5p | miR-34c | miR-29a |  |
| miR-7977 | miR-222-3p | miR-872 | miR-28 |  |
| miR-1915-3p | miR-4488 | miR-450a | miR-27a |  |
| miR-6088 | miR-103a-3p | miR-34b | miR-210 |  |
| Let-7a-5p | miR-320b | miR-214 | miR-20a |  |
| miR-3178 | miR-762 | miR-3593-3p | miR-20b |  |
| Let-7c-5p | miR-638 | miR-210 | miR-19a |  |
| miR-103a-3p | let-7b-5p | miR-34a | miR-19b |  |
| miR-4516 | miR-145-5p | miR-31 | miR-15b |  |
| miR-3656 | miR-1237-5p | miR-542-3p | miR-10b |  |
| miR-27a-3p | miR-31-5p | miR-290 | let-7e |  |
| miR-16-5p | miR-100-5p | miR-532-5p | let-7d |  |
| miR-23b-3p | miR-3656 | miR-25 | let-7a |  |
| miR-4488 | miR-3178 | miR-130a |  |  |
| Let7e-5p | miR-1469 | miR-31 |  |  |
| miR-638 | miR-4530 | miR-23b |  |  |
| miR-5787 | miR-125b-5p | miR-181a |  |  |
| miR-6727-5p | miR-23b-3p |  |  |  |
| miR-149-3p | miR-320c |  |  |  |
| miR-4508 | miR-107 |  |  |  |
| miR-1260b | miR-92a-3p |  |  |  |
| miR-214-3p | miR-8072 |  |  |  |
| **NA** | **80.0%** | **11.1%** | **14.6%** | **4.6%** |

**Supplementary References.**

Cheng, L., Zhang, K., Wu, S., Cui, M., & Xu, T. (2017). Focus on mesenchymal stem cell-derived exosomes: Opportunities and challenges in cell-free therapy. *Stem Cells International*. http://doi.org/10.1155/2017/6305295

Koniusz, S., Andrzejewska, A., Muraca, M., Srivastava, A. K., Janowski, M., & Lukomska, B. (2016). Extracellular Vesicles in Physiology, Pathology, and Therapy of the Immune and Central Nervous System, with Focus on Extracellular Vesicles Derived from Mesenchymal Stem Cells as Therapeutic Tools. *Frontiers in Cellular Neuroscience*, *10*. http://doi.org/10.3389/fncel.2016.00109

Lo Sicco, C., Reverberi, D., Balbi, C., Ulivi, V., Principi, E., Pascucci, L., … Tasso, R. (2017). Mesenchymal Stem Cell-Derived Extracellular Vesicles as Mediators of Anti-Inflammatory Effects : Endorsement of Macrophage Polarization. *STEM CELLS Translational Medicine*, *March*(6(3)), 1018–1028. http://doi.org/10.1002/sctm.16-0363

Sarvar, D. P., Shamsasenjan, K., & Akbarzadehlaleh, P. (2016). Mesenchymal stem cell-derived exosomes: New opportunity in cell-free therapy. *Advanced Pharmaceutical Bulletin*, *6*(3), 293–299. http://doi.org/10.15171/apb.2016.041

Wen, Z., Huang, W., Feng, Y., Cai, W., Wang, Y., Wang, X., … Wang, Y. (2014). MicroRNA-377 regulates mesenchymal stem cell-induced angiogenesis in ischemic hearts by targeting VEGF. *PLoS ONE*, *9*(9). http://doi.org/10.1371/journal.pone.0104666
